# CircFAM120B Blocks the Development of Colorectal Cancer by Activating TGF-Beta Receptor II Expression via Targeting miR-645

**DOI:** 10.3389/fcell.2021.682543

**Published:** 2021-07-26

**Authors:** You Yu, Xiao Lei

**Affiliations:** ^1^Department of General Surgery, Chongqing Bishan People’s Hospital, Chongqing, China; ^2^Department of General Surgery, The First Hospital Affiliated to AMU (Southwest Hospital), Chongqing, China

**Keywords:** circFAM120B, miR-645, TGFBR2, colorectal cancer, mechanism

## Abstract

Circular RNAs (circRNAs) are implicated in various human cancers, including colorectal cancer (CRC). The objective of this study was to investigate the function and regulatory mechanism of a novel circRNA, circFAM120B, in CRC development. The expression of circFAM120B, miR-645 and TGF-beta receptor II (TGFBR2) mRNA was detected by quantitative real-time polymerase chain reaction. Cellular biological functions, including cell proliferation, migration/invasion, and glycolysis metabolism, were assessed using CCK-8 assay, colony formation assay, transwell assay, and glycolysis stress test, respectively. Glycolysis progression was also monitored by lactate production and glucose consumption. The expression of glycolysis-related markers and TGFBR2 at the protein level was detected by western blot. The interaction between miR-645 and circFAM120B or TGFBR2 was predicted by bioinformatics analysis and verified by pull-down assay, dual-luciferase reporter assay and RIP assay. *In vivo* animal experiments were performed to further explore the function of circFAM120B. The expression of circFAM120B was decreased in CRC tissues and cells. CircFAM120B overexpression blocked CRC cell proliferation, migration/invasion, and glycolysis metabolism. MiR-645 was a target of circFAM120B, and miR-645 restoration reversed the effects of circFAM120B overexpression. In addition, TGFBR2 was a target of miR-645, and miR-645 inhibition-suppressed CRC cell proliferation, migration/invasion and glycolysis were restored by TGFBR2 knockdown. Moreover, circFAM120B activated the expression of TGFBR2 by targeting miR-645. TGFBR2 also blocked tumor growth *in vivo* by targeting the miR-645/TGFBR2 axis. CircFAM120B inhibited CRC progression partly by mediating the miR-645/TGFBR2 network, which explained the potential mechanism of circFAM120B function in CRC.

## Introduction

It is estimated that more than one million people worldwide suffer from colorectal cancer (CRC) every year worldwide (Torre et al., 2015; Testa et al., 2018). As one of the most common malignant tumors, CRC has gradually become the third cancer-related disease in males and second in females (Kuipers et al., 2015). Most CRC is defined as adenocarcinoma, which is subdivided into low-grade and high-grade according to the grade of the tumor (Testa et al., 2018). Recently, the development of targeted therapy and molecular markers has received great attention in metastatic CRC (Peluso et al., 2017; Price et al., 2018), benefiting to disease detection, tumor staging, patients’ response and outcomes after treatment (Taieb et al., 2019). This provides a beneficial strategy for the treatment and survival of patients. However, the pathogenesis of CRC is complex and needs further exploration.

Circular RNAs (circRNAs) are a group of the non-coding RNAs. CircRNAs are more stable compared to linear mRNA due to their closed-loop structures (Memczak et al., 2013). Besides, circRNAs can be easily checked in liquid biopsy of clinical specimens, including blood, serum, urine, and saliva (Zhang et al., 2018). With the bloom of RNA sequencing technology, dozens of circRNAs are shown to be differently expressed in tumor tissues relative to normal tissues, including CRC (Li et al., 2018), which hints that circRNAs are associated with cancer development. Increasing research has identified the biological functions of certain circRNAs on tumor growth and metastasis in CRC (Zhu et al., 2017; Ruan et al., 2019), but the literature of circRNA in CRC is still limited, remaining numerous functionally unknown circRNAs. CircRNA expression profile on Gene Expression Omnibus (GEO) database from a previous study provides several candidates that are dysregulated in CRC (Chen Z. et al., 2019). CircFAM120B, derived from the exon2–exon4 of FAM120B mRNA, is such a circRNA whose expression was notably decreased in CRC tissues. However, the function of circFAM120B in detail in CRC is not explored.

MicroRNAs (miRNAs) are also a class of non-coding RNAs. MiRNAs are well known for their regulatory potential by binding to 3′untranslated regions (3′UTRs) of mRNAs (Wolter et al., 2014). In addition, specific circRNAs harbor miRNA response elements (MREs) to target miRNAs and suppress miRNA expression (Cesana and Daley, 2013). Throughout the circRNA literature, the circRNA–miRNA–mRNA axis was constantly mentioned to enrich the regulatory networks in cancer progression (Chen L. Y. et al., 2019; Chen Z. et al., 2019; Li Y. et al., 2019). MiR-645 was reported to promote the oncogenesis of CRC (Guo et al., 2017), suggesting that miR-645 was a crucial regulator in CRC progression. More mechanisms of miR-645 function in CRC need to be explored.

The transforming growth factor-beta (TGF-beta) signaling pathway has indispensable effects on multiple cellular processes, such as cell proliferation, differentiation, cycle and apoptosis (Moustakas and Heldin, 2009). TGF-beta signaling pathway is implicated in the development of various types of cancer (Dong et al., 2012; Guo et al., 2012). TGF-beta receptor II (TGFBR2) was proved as a tumor suppressor in CRC (Li et al., 2015). The functional mechanism of TGFBR2 associated with miR-645 is not illustrated.

In this study, we validated the expression of circFAM120B and firstly investigated its function and mechanism in CRC, aiming to clarify the role of circFAM120B in CRC and broaden the insights into understanding CRC progression.

## Materials and Methods

### Specimen Collection

All specimens were collected from Chongqing Bishan People’s Hospital. A total of 50 CRC patients were recruited as subjects for this study. Tumor tissues and adjacent normal tissues were excised and used with the informed consent of each subject. All specimens were exposed to liquid nitrogen and stored at -80°C conditions. This study got the approval of the Ethical Committee of Chongqing Bishan People’s Hospital.

### Cell Lines

Colorectal cancer cell lines, including LoVo and HCT15, were purchased from ProCell Co., Ltd. (Wuhan, China) and maintained in matched special medium (ProCell Co., Ltd.). Normal colonic epithelial cells (NCM460) were purchased from BeNa Culture Collection (Beijing, China) and maintained in 90% Roswell Park Memorial Institute (RPMI-1640; Invitrogen, Carlsbad, CA, United States) containing 10% fetal bovine serum (FBS). All cells were cultured at 37°C conditions containing 5% CO_2_.

### Quantitative Real-Time Polymerase Chain Reaction

For circFAM120B and TGFBR2, total RNA isolated using Trizol reagent (Invitrogen) was then subjected to reverse transcription reaction using a High-Capacity cDNA Reverse Transcription Kit (Invitrogen) followed by qRT-PCR amplification reaction using the SYBR Green Master Mix (Invitrogen). For miR-645, reverse transcription and qRT-PCR amplification were conducted using a Bulge-Loop miRNA qRT-PCR Starter Kit (Ribobio Co., Ltd., Guangzhou, China). Glyceraldehyde-3-phosphate dehydrogenase (GAPDH) or U6 was used as an internal reference for circFAM120B and TGFBR2 expression or miR-645 expression, respectively. The final data were calculated using the 2^–ΔΔCt^ method. The primers used were listed in Table 1.

**TABLE 1:** The primer sequences for quantitative real-time polymerase chain reaction (qRT-PCR).

Name	Sequences (5′–3′)
CircFAM120B	F: 5′-CAGGCCTTCATTTACCGTCC-3′
	R: 5′-GAAAGGATCTGGAATGGTCATCT-3′
FAM120B	F: 5′-AATCCAGCCAATGCCATCCA-3′
	R: 5′-GAGAGCCAGATCCTCTGCG-3′
TGFBR2	F: 5′-GTAGCTCTGATGAGTGCAATGAC-3′
	R: 5′-CAGATATGGCAACTCCCAGTG-3′
GAPDH	F: 5′-TGGAAGGACTCATGACCACA-3′
	R: 5′-TTCAGCTCAGGGATGACCTT-3′
U6	F: 5′-CTCGCTTCGGCAGCACAT-3′
	R: 5′-AACGCTTCACGAATTTGCGT-3′
miR-645	F: 5′-CGCGCGTCTAGGCTGGTAC-3′
	R: 5′-AGTGCAGGGTCCGAGGTATT-3′
miR-561	F: 5′-GCGCGATCAAGGATCTTAAAC-3′
	R: 5′-AGTGCAGGGTCCGAGGTATT-3′
miR-576-5p	F: 5′-GCGCGATTCTAATTTCTCCAC-3′
miR-578	F: 5′-AGTGCAGGGTCCGAGGTATT-3′
	F: 5′-GCGCGCTTCTTGTGCTCTAG-3′
	F: 5′-AGTGCAGGGTCCGAGGTATT-3′
miR-602	F: 5′-GACACGGGCGACAGCTG-3′
	F: 5′-AGTGCAGGGTCCGAGGTATT-3′

### Actinomycin D Treatment

To test the stability of circFAM120B, LoVo, and HCT15 cells were probed with 2 μg/mL actinomycin D (Sigma, St. Louis, MO, United States) treatment for 4, 8, 12, and 24 h and then harvested at different time points. The stability of linear mRNA and circRNA was analyzed by qRT-PCR.

### Oligonucleotides, Plasmids, and Cell Transfection

CircFAM120B was overexpressed using pLO5-ciR vector, and pLO5-ciR-circFAM120B plasmid (oe-circFAM120B), which was constructed by Genepharma (Shanghai, China) with single vector (vector) as a control. MiR-645 mimics and inhibitors (miR-645 and anti-miR-645) were purchased from Ribobio Co., Ltd., with miR-NC or anti-miR-NC as a negative control. TGFBR2 was downregulated using small interference RNA targeting TGFBR2 (si-TGFBR2) (Genepharma) with si–NC as a negative control. The oligonucleotides and plasmids were transfected into LoVo and HCT15 cells for functional analyses using Lipofectamine 3000 (Invitrogen).

### CCK-8 Assay

Colorectal cancer cells with diverse transfection were planted into 96-well plates (2,000 cells/well). At the indicated time points (24, 48, and 72 h), 10 μL CCK-8 reagent (Sangon Biotech, Shanghai, China) was pipetted into each well, incubating for another 2 h. The absorbance at 450 nm was detected using iMark microplate reader (Bio-Rad, Hercules, CA, United States).

### Colony Formation Assay

Colorectal cancer cells with diverse transfection were planted into six-well plates (1,000 cells/well) and cultured in an incubator at 37°C, to allow colony growth. Colonies were observed every 3 days, and after 2 weeks, colonies were fixed with paraformaldehyde and stained with 0.1% crystal violet and investigated under a microscope (Olympus, Tokyo, Japan).

### Transwell Assay

Transwell chambers (BD Bioscience, San Jose, CA, United States) were used to monitor cell migration and invasion. CRC cells with diverse transfection were collected and resuspended 100 μL culture medium. Subsequently, cells in fresh culture medium were added into the upper chambers for migration analysis or added into the upper chambers pre-coated with Matrigel (BD Bioscience) for invasion analysis. At the same time, the lower chambers were supplemented with culture medium to induce cell migration or invasion. After 24 h, cells in the lower surface were fixed with paraformaldehyde and stained with 0.1% crystal violet. The results were monitored under a microscope (Olympus) with magnification of 100×.

### Extracellular Acidification Rate Test

Extracellular Acidification Rate Test (ECAR) was detected by Seahorse Bioscience XF-24 Extracellular Flux Analyzer (Seahorse Bioscience, North Billerica, MA, United States) to monitor metabolic alternations *in vitro*. In brief, CRC cells with diverse transfection were cultured in XF24-well microplates (20,000 cells/well) (Seahorse Bioscience) for 24 h. Then, cells were incubated with unbuffered medium followed by sequential addition of 10 mM glucose, 1 μM oligomycin (OM), and 80 mM 2-deoxyglucose (2-DG) from a Seahorse XF glycolysis stress test Kit (Seahorse Bioscience) according to the directions. ECAR was shown as mpH/min. Each sample was tested in triplicate.

### Lactate Production and Glucose Consumption Detection

Lactate assay Kit (colorimetric; ab65330) and Glucose Assay Kit (colorimetric; ab136955) were purchased from Abcam (Cambridge, MA, United States). All experimental procedures were performed following the protocols to examine lactate production and glucose consumption.

### Western Blot

Total proteins were isolated by 12% sodium dodecyl sulfate-polyacrylamide gel electrophoresis (SDS-PAGE) and transferred into polyvinylidene difluoride (PVDF) membranes (Bio-Rad). After blocking by non-fat milk, the membranes were probed with the primary antibodies, including anti-Hexokinase 2 (HK2) (ab104836; Abcam), anti-Lactate dehydrogenase A (LDHA) (ab92903; Abcam), anti-TGFBR2 (ab204100; Abcam) and anti-GAPDH (ab8245; Abcam) at 4°C overnight, followed by the incubation of the secondary antibodies (ab205719; Abcam) at room temperature for 1 h. The protein signals were viewed using the enhanced chemiluminescence kit (Sangon Biotech).

### Bioinformatics Analysis

Bioinformatics tool Circular RNA Interactome^1^ was employed to predict the potential target miRNAs of circFAM120B. Another bioinformatics tool Targetscan^2^ was employed to predict the potential target mRNAs of miR-645.

### RNA Pull-Down Assay

Biotinylated-circFAM120B probe and oligo probe (negative control) were synthesized by Ribobio Co., Ltd., and incubated with Streptavidin C1-conjugated Dynabeads (Invitrogen). CRC cells (1 × 10^7^) were harvested and lysed using lysis buffer (Thermo Fisher Scientific, Waltham, MA, United States). Cell lysates were incubated with probe-coated beads at 4°C overnight. The RNA complexes bound to the beads were eluted, purified and subjected to qRT-PCR.

### Dual-Luciferase Reporter Assay

The information of luciferase reporter vector (PGL4; Promega, Madison, WI, United States) was shown in Supplementary Figure 3C. PGL4 containing circFAM120B wild-type sequence (harboring miR-645 binding sites) or circFAM120B mutant-type sequence (harboring mutated miR-645 binding sites) were constructed by Genepharma, and fusion plasmids were named as circFAM120B WT and circFAM120B MUT. In the same way, fusion plasmids, TGFBR2 3′UTR WT and TGFBR2 3′UTR MUT, were also constructed. To validate the interaction between circFAM120B and miR-645, LoVo, and HCT15 cells were transfected with miR-645 and circFAM120B WT or circFAM120B MUT, with miR-NC as a control. To validate the interaction between TGFBR2 and miR-645, LoVo, and HCT15 cells were transfected with miR-645 and TGFBR2 3′UTR WT or TGFBR2 3′UTR MUT, with miR-NC as a control. Luciferase activity in cells with cotransfection was examined at 48 h post-transfection using the dual-luciferase assay system (Promega).

### RIP Assay

The Magna RIP Kit (Millipore, Billerica, MA, United States) was applied here for RIP assay. In brief, LoVo and HCT115 cells were lysed and nexted exposed to Ago2 antibody-conjugated or IgG antibody-conjugated magnetic beads. RNA complex on magnetic beads was eluted to isolate total RNA. Finally, qRT-PCR assay was conducted to detect the expression of indictors.

### Animal Experiments

The experimental procedures were approved by the Animal Care and Use Committee of Chongqing Bishan People’s Hospital. BALB/c nude mice (*n* = 12; male; weight: 20–25 g; 4–6 weeks-old) were purchased from Shanghai SLAC Laboratory Animal Co., Ltd. (Shanghai, China) and assigned into two groups (*n* = 6 per group). LoVo cells were transfected with oe-circFAM120B or vector and then subcutaneously implanted into the right groin of mice. All mice were regularly kept and observed 1 week after implant. Subsequently, tumor volume was measured and recorded once a week. After 5 weeks, all mice were sacrificed, and tumor tissues were collected for further experiments.

### Immunohistochemical Staining Analysis

Tumor tissues were fixed, embedded in paraffin and cut into 5-μm-thick sections. Afterward, the sections were deparaffinized, rehydrated and received antigen retrieve. The sections were incubated with the primary antibody targeting Ki67 (ab92742) at 4°C overnight and then incubated with the secondary antibody (ab205718). Tissue staining was performed using 3,3′-diaminobenzidine (DAB) Substrate Kit (Solarbio, Beijing, China).

### Statistical Analysis

All statistical analyses were performed using GraphPad Prism 5.0 (GraphPad Software, La Jolla, CA, United States). Student’s *t*-test was utilized to evaluate the differences between two groups, and analysis of variance was utilized to analyze the differences among multiple groups. All experiments were repeated at least three times, and the final data were presented as mean ± standard deviation. Differences were considered to be significant when *P* < 0.05.

## Results

### CircFAM120B Was Downregulated in CRC Tissues and Cells

**FIGURE 1:**
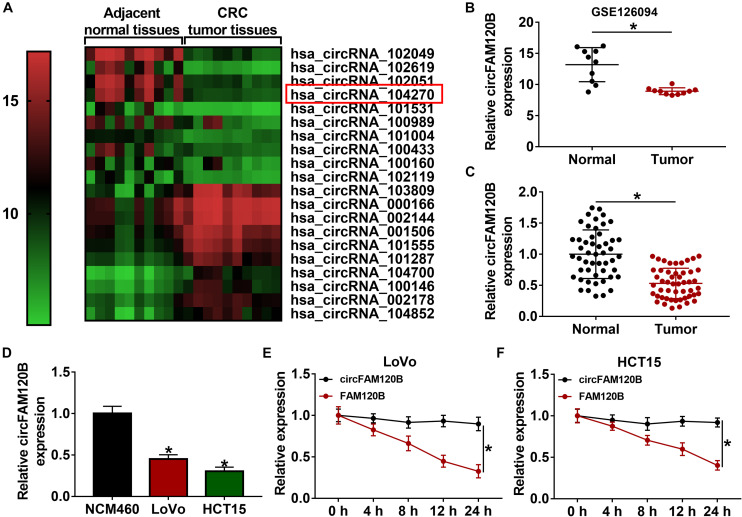
CircFAM120B was downregulated in colorectal cancer (CRC) tissues and cells. **(A,B)** A microarray profile revealed that circFAM120B was a differently expressed circRNA that was significantly downregulated in CRC tissues (*n* = 10). **(C)** The expression of circFAM120B in tumor tissues (*n* = 50) and normal tissues (*n* = 50) was detected by quantitative real-time polymerase chain reaction (qRT-PCR). **(D)** The expression of circFAM120B in LoVo, HCT15, and NCM460 cells was detected by qRT-PCR. **(E,F)** LoVo and HCT15 cells were exposed to actinomycin D to test the stability of circFAM120B. *P < 0.05.

The data of circRNA expression profile were obtained from the GEO dataset (GEO accession: GSE126094^3^). Heat map was depicted to observe the top 10 circRNAs which were significantly upregulated or downregulated in CRC tissues (*n* = 10) compared to adjacent normal tissues (*n* = 10), and circFAM120B (has_circRNA_104270) was shown to be notably downregulated in CRC tissues (Figures 1A,B). CircFAM120B was generated from the exon2–4 of precursor FAM120B mRNA in a “back-splicing” way, and the schematic was depicted to illustrate the formation of circFAM120B in Supplementary Figure 1A. In collected clinical specimens, the expression of circFAM120B was significantly declined in CRC tumor tissues (*n* = 50) compared with that in normal tissues (*n* = 50) (Figure 1C). Meanwhile, the expression of circFAM120B was also decreased in several CRC cell lines (LoVo, HCT15, SW620, HCT116, HCT8, and CACO2) compared to normal colonic epithelial cells (NCM460) (Supplementary Figure 1B and Figure 1D), and circFAM120B showed lower expression in LoVo and HCT15 cells compared to other cell lines. In stability test, the data showed that circFAM120B was more stable than FAM120B abundance in LoVo and HCT15 cells treated with actinomycin D (Figures 1E,F). In short, these data indicated that circFAM120B was aberrantly downregulated in CRC and might regulate CRC growth.

### Enhanced Expression of circFAM120B Blocked CRC Cell Proliferation, Migration/Invasion, and Glycolysis Metabolism

**FIGURE 2:**
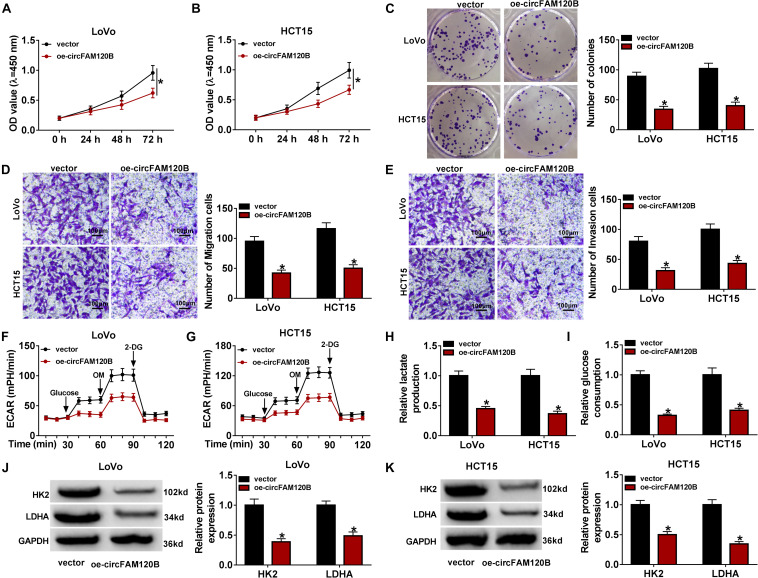
CircFAM120B overexpression weakened cell proliferation, migration/invasion and glycolysis. In circFAM120B-overexpressed LoVo and HCT15 cells, **(A–C)** cell proliferation was assessed by CCK-8 assay and colony formation assay. **(D,E)** Cell migration and invasion were determined by transwell assay (magnification: 100×). **(F,G)** Extracellular acidification rate (ECAR) was measured by glycolysis stress test to evaluate glycolysis metabolism. **(H,I)** Lactate production and glucose consumption were quantified to assess glycolysis progression using corresponding kits. **(J,K)** Glycolysis-related markers, HK2 and LDHA, were quantified by western blot to observe glycolysis. *P < 0.05.

For functional analyses, we explored the role of circFAM120B on proliferation, migration/invasion and metabolism. The examination of circFAM120B overexpression construct showed that circFAM120B expression was markedly enhanced in LoVo and HCT15 cells transfected with oe-circFAM120B compared to vector transfection or no-transfection (control) (Supplementary Figure 2A). Cell proliferation was assessed by CCK-8 assay and colony formation assay. The results displayed that the OD value at 450 nm and the number of colonies were markedly impaired in LoVo and HCT15 cells transfected with oe-circFAM120B (Figures 2A–C), suggesting that cell proliferation was suppressed by circFAM120B overexpression. Transwell migration and invasion assay introduced that the number of migrated and invaded cells was notably reduced by circFAM120B overexpression (Figures 2D,E). Cellular energy metabolism was monitored by ECAR value of glycolysis stress test. The data exhibited that the value of ECAR was prominently declined in LoVo and HCT15 cells transfected with oe-circFAM120B compared to vector with the ordinal addition of glucose, OM and 2-DG (Figures 2F,G). Besides, the production of lactate and the consumption of glucose were suppressed by circFAM120B overexpression (Figures 2H,2I). In addition, the expression of glycolysis-related proteins, including HK2 and LDHA, was also weakened in cells with circFAM120B overexpression (Figures 2J,K). These data suggested that circFAM120B blocked the malignant development of CRC via suppressing CRC cell proliferation, migration/invasion and glycolysis metabolism.

### MiR-645, a Target of circFAM120B, Was Upregulated in CRC Tissues and Cells

**FIGURE 3:**
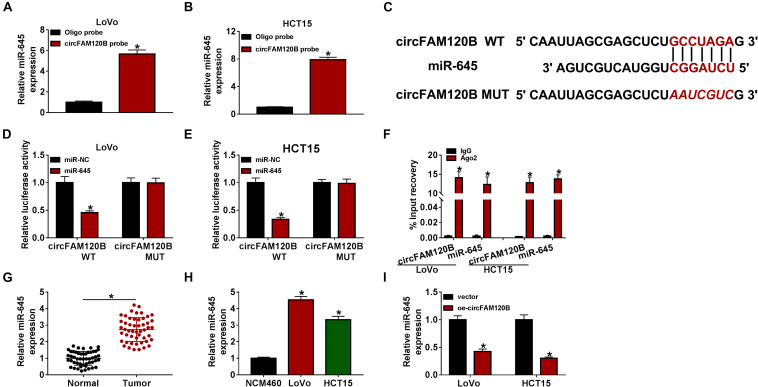
MiR-645 was a target of circFAM120B. **(A,B)** Biotinylated-circFAM120B probe could significantly enrich the level of miR-645. **(C)** Wild-type circFAM120B sequence containing miR-645 binding sites was mutated to generate mutant-type circFAM120B sequence for dual-luciferase reporter assay. **(D,E)** Dual-luciferase reporter assay was performed to further validate the relationship between miR-645 and circFAM120B. **(F)** RIP assay was performed to validate the relationship between miR-645 and circFAM120B. **(G)** The expression of miR-645 in tumor tissues (*n* = 50) and normal tissues (*n* = 50) was detected by qRT-PCR. **(H)** The expression of miR-645 in LoVo, HCT15, and NCM460 cells was detected by qRT-PCR. **(I)** The expression of miR-645 in LoVo and HCT15 cells with circFAM120B overexpression was detected by qRT-PCR. *P < 0.05.

To validate whether circFAM120B exerted functions by targeting downstream miRNAs, we screened and verified the potential miRNAs targeted by circFAM120B. A biotinylated-circFAM120B probe was designed to screen the putative target miRNAs, and the probe was able to substantially pull-down circFAM120B in LoVo and HCT15 cells. Besides, the pull-down efficiency was increased in cells with circFAM120B overexpression (Supplementary Figures 3A,B). MiR-645 was richly pulled down by circFAM120B probe in both LoVo and HCT15 cells (Figures 3A,B). Besides, the expression of miR-645 showed the greatest decrease in LoVo and HCT15 cells with circFAM120B overexpression compared to other putative target miRNAs of circFAM120B (Supplementary Figures 3D,E). Hence, miR-645 was chosen for the following assays. To perform dual-luciferase reporter assay, circFAM120B mutation sequence (binding site mutation) was designed according to its wild sequence (Figure 3C). The efficiency of miR-645 mimic was checked, and the data exhibited that miR-645 expression was strikingly elevated in LoVo and HCT15 cells with miR-645 transfection (Supplementary Figure 2B). Dual-luciferase reporter assay presented that the luciferase activity was pronouncedly decreased in LoVo and HCT15 cells cotransfected with circFAM120B WT but not circFAM120B MUT and miR-645 (Figures 3D,E). RIP assay showed that both circFAM120B and miR-645 could be abundantly enriched in the Ago2-RIP group compared with that in the IgG-RIP group (Figure 3F). The expression of miR-645 was notably reinforced in CRC tumor tissues (*n* = 50) compared to normal tissues (*n* = 50) (Figure 3G). Also, the expression of miR-645 was elevated in LoVo and HCT15 cells compared to NCM460 cells (Figure 3H). Moreover, the expression of miR-645 was significantly declined in LoVo and HCT15 cells with circFAM120B overexpression (Figure 3I). All findings pointed out that miR-645 was a target of circFAM120B.

### MiR-645 Overexpression Reversed the circFAM120B Overexpression-Induced Inhibition of CRC Development *in vitro*

**FIGURE 4:**
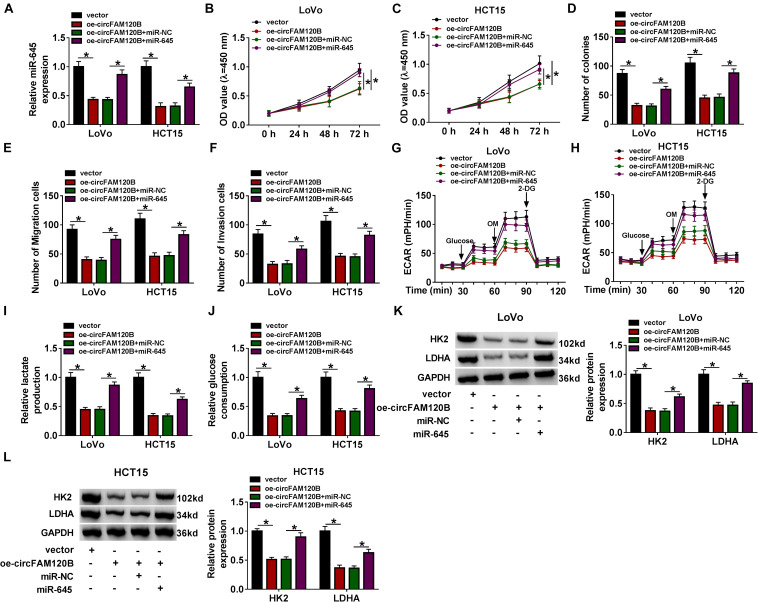
CircFAM120B blocked CRC progression *in vitro* by targeting miR-645. LoVo and HCT15 cells were transfected with oe-circFAM120B alone or oe-circFAM120B + miR-645, with vector or oe-circFAM120B + miR-NC as a control. **(A)** The expression of miR-645 in these transfected cells was detected by qRT-PCR. **(B,C)** Cell proliferation was assessed by CCK-8 assay. **(D)** Cell proliferation was assessed by colony formation assay. **(E,F)** Cell migration and invasion were monitored by transwell assay (magnification: 100×). **(G,H)** Glycolysis was evaluated by ECAR using glycolysis stress test. **(I,J)** Lactate production and glucose consumption were quantified to monitor glycolysis. **(K,L)** The expression of HK2 and LDHA was quantified by western blot. *P < 0.05.

To figure out whether circFAM120B-mediated inhibition of CRC progression was dependent on the regulation of miR-645, we used oe-circFAM120B + miR-645 to transfect CRC cells. QRT-PCR analysis indicated that the expression of miR-645 impaired by oe-circFAM120B transfection was notably recovered by oe-circFAM120B + miR-645 transfection in LoVo and HCT15 cells (Figure 4A). OD value and colony number were lessened by oe-circFAM120B transfection alone, and miR-645 reintroduction largely recovered OD value and colony number in cells transfected with oe-circFAM120B (Figures 4B–D). Cell migration and invasion were inhibited by oe-circFAM120B transfection alone but partly reinforced in cells transfected with oe-circFAM120B + miR-645 (Figures 4E,F). Glycolysis rate and glycolysis capacity were blocked by circFAM120B overexpression, and miR-645 restoration partly promoted glycolysis rate and glycolysis capacity in cells with circFAM120B overexpression (Figures 4G,H). Lactate production and glucose consumption impaired in cells transfected with oe-circFAM120B alone were substantially strengthened in cells cotransfected with oe-circFAM120B + miR-645 (Figures 4I,J). The expression levels of HK2 and LDHA were lessened by oe-circFAM120B transfection but largely enhanced in cells by oe-circFAM120B + miR-645 cotransfection in LoVo and HCT15 cells (Figures 4K,L). Our results suggested that circFAM120B blocked the progression of CRC by targeting miR-645.

### TGFBR2 Was a Direct Target of miR-645

**FIGURE 5:**
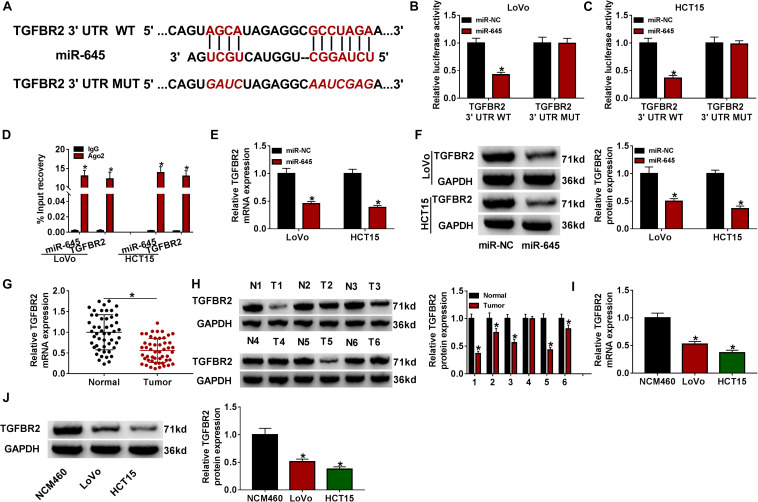
TGFBR2 was a target of miR-645. **(A)** Wild-type TGFBR2 3′UTR sequence containing miR-645 binding sites was mutated to generate mutant-type TGFBR2 3′UTR sequence for dual-luciferase reporter assay. **(B,C)** Dual-luciferase reporter assay was conducted to verify the interaction between miR-645 and TGFBR2. **(D)** RIP assay was conducted to verify the interaction between miR-645 and TGFBR2. **(E,F)** The expression of TGFBR2 suppressed by miR-645 restoration was detected by qRT-PCR and western blot. **(G,H)** The expression of TGFBR2 in tumor tissues (*n* = 50) and normal tissues (*n* = 50) was detected by qRT-PCR and western blot. **(I,J)** The expression of TGFBR2 in LoVo, HCT15 and NCM460 cells was detected by qRT-PCR and western blot. *P < 0.05.

To further clarify the mechanisms by which circFAM120B regulated CRC progression, we analyzed the potential target mRNAs of miR-645. As displayed in Figure 5A, miR-645 combined with the 3′UTR of TGFBR2 mRNA through several binding sites (Figure 5A). TGFBR2 3′UTR mutant fragment was designed according to its wild fragment. As displayed in Figures 5B,C, luciferase activity of LoVo and HCT15 cells transfected with TGFBR2 3′UTR WT was decreased when miR-645 was overexpressed in cells. RIP assay indicated that both miR-645 and IGFBR2 could be significantly enriched in the Ago2-RIP group compared to IgG (Figure 5D). The expression of TGFBR2 was strikingly impaired in miR-645-overexpressed LoVo and HCT15 cells at both mRNA and protein levels (Figures 5E,F). Besides, the expression of TGFBR2 was aberrantly declined in CRC tumor tissues at both mRNA and protein levels (Figures 5G,H). Likewise, the expression of TFGBR2 was also decreased in LoVo and HCT15 cells compared to NCM460 cells at both mRNA and protein levels (Figures 5I,J). In short, TGFBR2 was a target of miR-645, and it was poorly expressed in CRC tissues and cells.

### TGFBR2 Knockdown Reversed the miR-645 Inhibition-Induced Inhibition of CRC Progression *in vitro*

**FIGURE 6:**
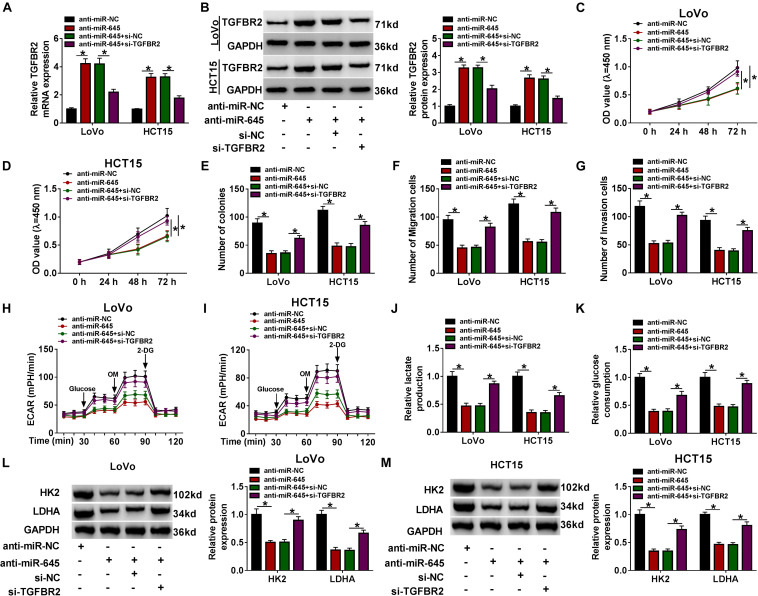
miR-645 promoted CRC progression *in vitro* by mediating TGFBR2. LoVo and HCT15 cells were transfected with anti-miR-645 or anti-miR-645 + si-TGFBR2, with anti-miR-NC or anti-miR-645 + si-NC as the corresponding control. **(A,B)** The expression of TGFBR2 was examined using qRT-PCR and western blot. **(C–E)** Cell proliferation was investigated using CCK-8 assay and colony formation assay. **(F,G)** Cell migration and invasion were monitored by transwell assay (magnification: 100×). **(H,I)** ECAR was measured by glycolysis stress test. **(J,K)** Lactate production and glucose consumption were detected using the corresponding kits. **(L,M)** The expression of HK2 and LDHA was measured by western blot. *P < 0.05.

Subsequently, we functionally investigated the interaction between miR-645 and TGFBR2. The efficiency of miR-645 inhibitor and si-TGFBR2 was checked. The data displayed that miR-645 expression was notably declined in LoVo and HCT15 cells transfected with anti-miR-645 (Supplementary Figure 2C), and TGFBR2 expression was strikingly lessened in cells transfected with si-TGFBR2 at both mRNA and protein levels (Supplementary Figures 2D,E). LoVo and HCT15 cells were transfected with anti-miR-645 or anti-miR-645 + si-TGFBR2, with anti-miR-NC or anti-miR-645 + si-NC as the separate control. The expression of TGFBR2 was promoted in cells transfected with anti-miR-645 compared to anti-miR-NC but was weakened in cells transfected with anti-miR-645 + si-TGFBR2 compared to anti-miR-645 + si-NC at the protein level (Figures 6A,B). CCK-8 assay and colony formation assay showed that cell proliferation was remarkably suppressed by miR-645 inhibition, while TGFBR2 knockdown largely recovered cell proliferation in cells treated with miR-645 inhibition (Figures 6C–E). Besides, the capacities of migration and invasion were repressed in cells transfected with anti-miR-645, while these capacities were partly enhanced in cells transfected with anti-miR-645 + si-TGFBR2 (Figures 6F,G). The value of ECAR was depressed in cells transfected with anti-miR-645, while ECAR value was strengthened in cells transfected with anti-miR-645 + si-TGFBR2 (Figures 6H,I). As expected, the production of lactate and the consumption of glucose were impaired in cells with miR-645 inhibition but heightened in cells with miR-645 inhibition and TGFBR2 knockdown (Figures 6J,K). The expression of HK2 and LDHA suppressed by miR-645 inhibition was also recovered by miR-645 inhibition plus TGFBR2 knockdown (Figures 6L,M). Collectively, miR-645 promoted the development of CRC *in vitro* by targeting TGFBR2.

### CircFAM120B Suppressed the Expression of miR-645 to Upregulate TGFBR2

**FIGURE 7:**
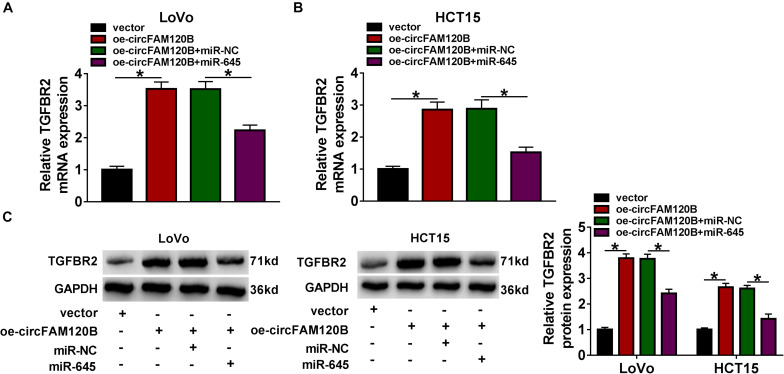
CircFAM120B activated the expression of TGFBR2 by targeting miR-645. In LoVo and HCT15 cells transfected with oe-circFAM120B, vector, oe-circFAM120B + miR-645 or oe-circFAM120B + miR-NC, the expression of TGFBR2 was detected by **(A,B)** qRT-PCR and **(C)** western blot. *P < 0.05.

Interestingly, the expression of TGFBR2 was predominantly enhanced in LoVo and HCT15 cells transfected with oe-circFAM120B compared to vector, while the expression of TGFBR2 was substantially decreased in cells transfected with oe-circFAM120B + miR-645 compared to circFAM120B + miR-NC at both mRNA and protein levels (Figures 7A–C). These data highlighted that circFAM120B regulated TGFBR2 expression by targeting miR-645.

### Enhanced Expression of circFAM120B Inhibited CRC Tumor Growth *in vivo*

**FIGURE 8:**
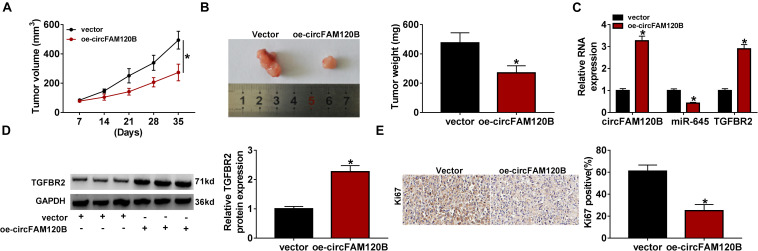
CircFAM120B blocked tumor growth *in vivo* by regulating miR-645 and TGFBR2. **(A)** Tumor volume was measured once a week. **(B)** After 35 days, tumor tissues were removed for weighting. **(C)** The expression of circFAM120B, miR-645, and TGFBR2 mRNA in removed tumor tissues was detected by qRT-PCR. **(D)** The expression of TGFBR2 at the protein level was detected by western blot. **(E)** The enrichment of Ki67 in tumor tissues was detected by immunohistochemical (IHC) staining analysis. *P < 0.05.

Animal experiments were performed to explore the role of circFAM120B *in vivo*. LoVo cells transfected with oe-circFAM120B or vector were implanted into the right groin of nude mice. As shown in Figures 8A,B, overexpression of circFAM120B led to decreased tumor volume and tumor weight, hinting that circFAM120B overexpression blocked tumor growth. Moreover, qRT-PCR data suggested that the expression of circFAM120B and TGFBR2 mRNA was reinforced, while the expression of miR-645 was lessened in the oe-circFAM120B mice group compared with that in the vector mice group (Figure 8C). Western blot data showed that the protein level of TGFBR2 was also elevated in the oe-circFAM120B group (Figure 8D). Moreover, IHC staining assay displayed that the abundance of Ki67 in tumor tissues was notably decreased by circFAM120B overexpression (Figure 8E). We obtained that circFAM120B regulated the miR-645/TGFBR2 pathway to limit tumor growth *in vivo*.

## Discussion

In our present study, we detected that circFAM120B expression was aberrantly declined in CRC tissues and cells. CircFAM120B overexpression inhibited several malignant cell behaviors, including cell proliferation, migration, invasion, and glycolysis metabolism. Further mechanism analyses illustrated that circFAM120B played these functions in CRC partly through circFAM120B-mediated miR-645/TGFBR2 network. These findings enriched the role of circFAM120B in CRC and broadened insights into understanding CRC pathogenesis.

Currently, the understanding of circRNAs in cancer provides new opportunities in cancer-related therapy (Su et al., 2019). To date, numerous CRC-involved circRNAs are functionally identified. For instance, circ_0003906 was downregulated in CRC tissues and cells, and low expression of circ_0003906 was linked to lymphatic metastasis (Zhuo et al., 2017). High-throughput sequencing uncovered that circVAPA was overexpressed in CRC tissues, and downregulated circVAPA repressed CRC cell proliferation, metastasis and invasion (Li X. N. et al., 2019). Circ_0002138 held a weak level in CRC tissues, and enriched circ_0002138 blocked CRC cell proliferation and viability (Ruan et al., 2019). These circRNAs in CRC could function as either oncogene or tumor suppressor. In our views, we reported a circRNA, circFAM120B, which was insufficiently expressed in CRC tissues by a previous circRNA expression profile analysis (Chen Z. et al., 2019). Interestingly, circFAM120B was also identified to be downregulated in breast cancer tissues by microarray analysis (Afzali and Salimi, 2019), implying that circFAM120B might be widely deficiently expressed in various cancers. Our expression data by qRT-PCR were consistent with these consequences, exhibiting that circFAM120B was strikingly downregulated in CRC tissues and cells. For functional analysis, we demonstrated that forced expression of circFAM120B inhibited CRC cell proliferation, migration/invasion and glycolysis metabolism *in vitro*, and blocked tumor growth *in vivo*. For mechanism analysis, we proposed that circFAM120B targeted miR-645 to play its anti-tumor effects.

MiR-645 was located at chromosome 20q13.13 and was highly expressed in CRC tissues, and miR-645 deficiency suppressed CRC cell proliferation and drug sensitivity (Guo et al., 2017). Besides, miR-645 enrichment induced CRC cell migration, invasion and epithelial-mesenchymal transition (EMT) (Li et al., 2020). Similarly, our data presented that miR-645 expression was also highly elevated in CRC tissues and cells. Inhibition of miR-645 restrained cell proliferation, migration/invasion and glycolysis metabolism of CRC *in vitro*. Moreover, miR-645 restoration reversed the anti-tumor effects of circFAM120B overexpression, suggesting that circFAM120B blocked CRC malignant behaviors by targeting miR-645. The carcinogenesis of miR-645 was also reported in osteosarcoma, renal clear cell carcinoma, and head and neck cancer (Sun et al., 2015; Chen et al., 2017; Jiao et al., 2018), indicating that miR-645 was an oncogenic driver in diverse cancers.

Additionally, our data disclosed that miR-645 directly bound to TGFBR2 3′UTR. Previous studies also stated that miR-645 bound to downstream target mRNAs (EFNA5) to regulate CRC development (Li et al., 2020), and TGFBR2 could also be acted as a target of miR-135b and involved in CRC cell proliferation and apoptosis through the miR-135b/TGFBR2 pathway (Li et al., 2015). In addition, the inactivation of TGFBR2 promoted CRC carcinogenesis, such as proliferation (Miguchi et al., 2016). Previous findings all maintained that TGFBR2 played an anti-tumor role in CRC. Our present data proved that the expression of TGFBR2 was decreased in CRC tissues and cells. TGFBR2 function as a target of miR-645, and its knockdown abolished the functional effects of miR-645 inhibition.

## Conclusion

Collectively, circFAM120B, aberrantly downregulated in CRC tissues and cells, might mediate the development and aggravation of CRC. CircFAM120B overexpression suppressed CRC cell proliferation, migration/invasion and glycolysis metabolism and also blocked tumor growth *in vivo* by targeting the miR-645/TGFBR2 axis. Our study enriches the role and functional mechanism of circFAM120B in CRC and provides potential new strategies for CRC treatment. However, there are still limitations in our present study. For example, the miR-645/TGFBR2 axis is the only one of the regulatory networks of circFAM120B in CRC, and more related pathways should be identified in further work. Besides, the canonical oncogenic signaling pathway that is involved in the circFAM120B/miR-645/TGFBR2 axis should be investigated. In this way, the role of circFAM120B in CRC will be clearer.

## Data Availability Statement

The raw data supporting the conclusions of this article will be made available by the authors, without undue reservation.

## Ethics Statement

The studies involving human participants were reviewed and approved by Chongqing Bishan People’s Hospital. The patients/participants provided their written informed consent to participate in this study. The animal study was reviewed and approved by Chongqing Bishan People’s Hospital.

## Author Contributions

Both authors contributed to data analysis, drafting or revising the article, gave final approval of the version to be published, and agreed to be accountable for all aspects of the work.

## Conflict of Interest

The authors declare that the research was conducted in the absence of any commercial or financial relationships that could be construed as a potential conflict of interest.

## Publisher’s Note

All claims expressed in this article are solely those of the authors and do not necessarily represent those of their affiliated organizations, or those of the publisher, the editors and the reviewers. Any product that may be evaluated in this article, or claim that may be made by its manufacturer, is not guaranteed or endorsed by the publisher.

## Footnotes

1 https://circinteractome.nia.nih.gov/

2 http://www.targetscan.org/vert_72/

3 https://www.ncbi.nlm.nih.gov/geo/geo2r/?acc=GSE126094

## Supplementary Material

Supplementary Figure 1 The schematic of circFAM120B production, and the expression of FAM120B in CRC cell lines. **(A)** CircFAM120B was derived from the exon2–exon4 of FAM120B. **(B)** The expression of circFAM120B in NCM460 cells and colorectal cancer CRC cell lines was detected by qRT-PCR. ^∗^*P* < 0.05.

Supplementary Figure 2 The efficiency of different constructs. **(A)** The efficiency of oe-circFAM120B was checked by qRT-PCR. **(B)** The efficiency of miR-645 mimic was checked using qRT-PCR. **(C)** The efficiency of miR-645 inhibitor was checked using qRT-PCR. **(D,E)** The efficiency of si-TGFBR2 was checked using qRT-PCR and western blot. ^∗^*P* < 0.05.

Supplementary Figure 3 The screening of miR-645. **(A,B)** The pull-down efficiency of circFAM120B probe was increased in LoVo and HCT15 cells with circFAM120B overexpression. **(C)** The information of luciferase reporter vector (PGL4). **(D,E)** The expression of putative target miRNAs of circFAM120B predicted by the bioinformatics tool was measured in LoVo and HCT15 cells with circFAM120B overexpression using qRT-PCR. ^∗^*P* < 0.05.
