# Supplementary figures and images for: CircFAM120B Blocks the Development of Colorectal Cancer by Activating TGF-Beta Receptor II Expression via Targeting miR-645
Source: Front Cell Dev Biol. 2021 Jul 26;9:682543. doi: 10.3389/fcell.2021.682543 (PMC8350741; doi:10.3389/fcell.2021.682543)

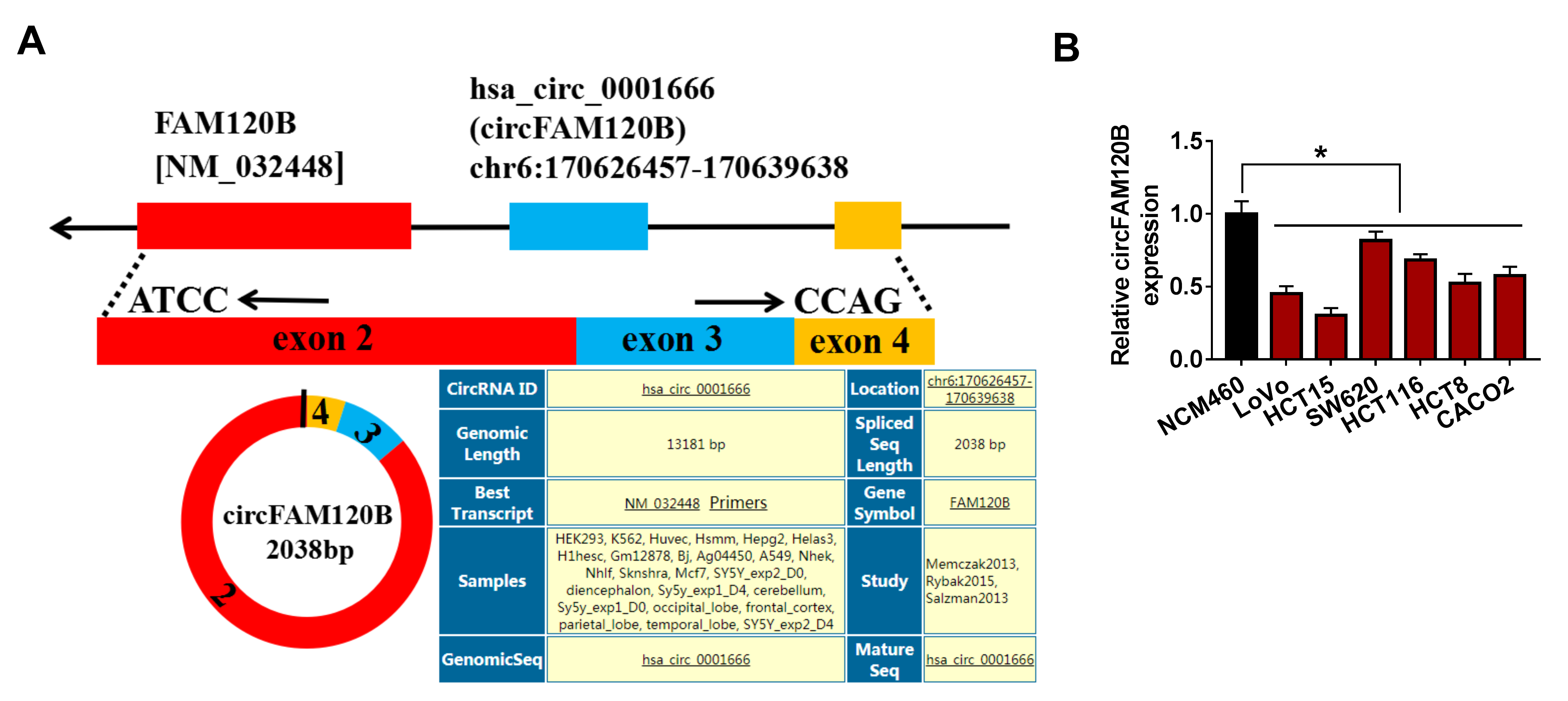

Supplement: Supplementary Figure 1 — The schematic of circFAM120B production, and the expression of FAM120B in CRC cell lines. (A) CircFAM120B was derived from the exon2–exon4 of FAM120B. (B) The expression of circFAM120B in NCM460 cells and colorectal cancer CRC cell lines was detected by qRT-PCR. ∗P < 0.05. [file Image_1.TIF]

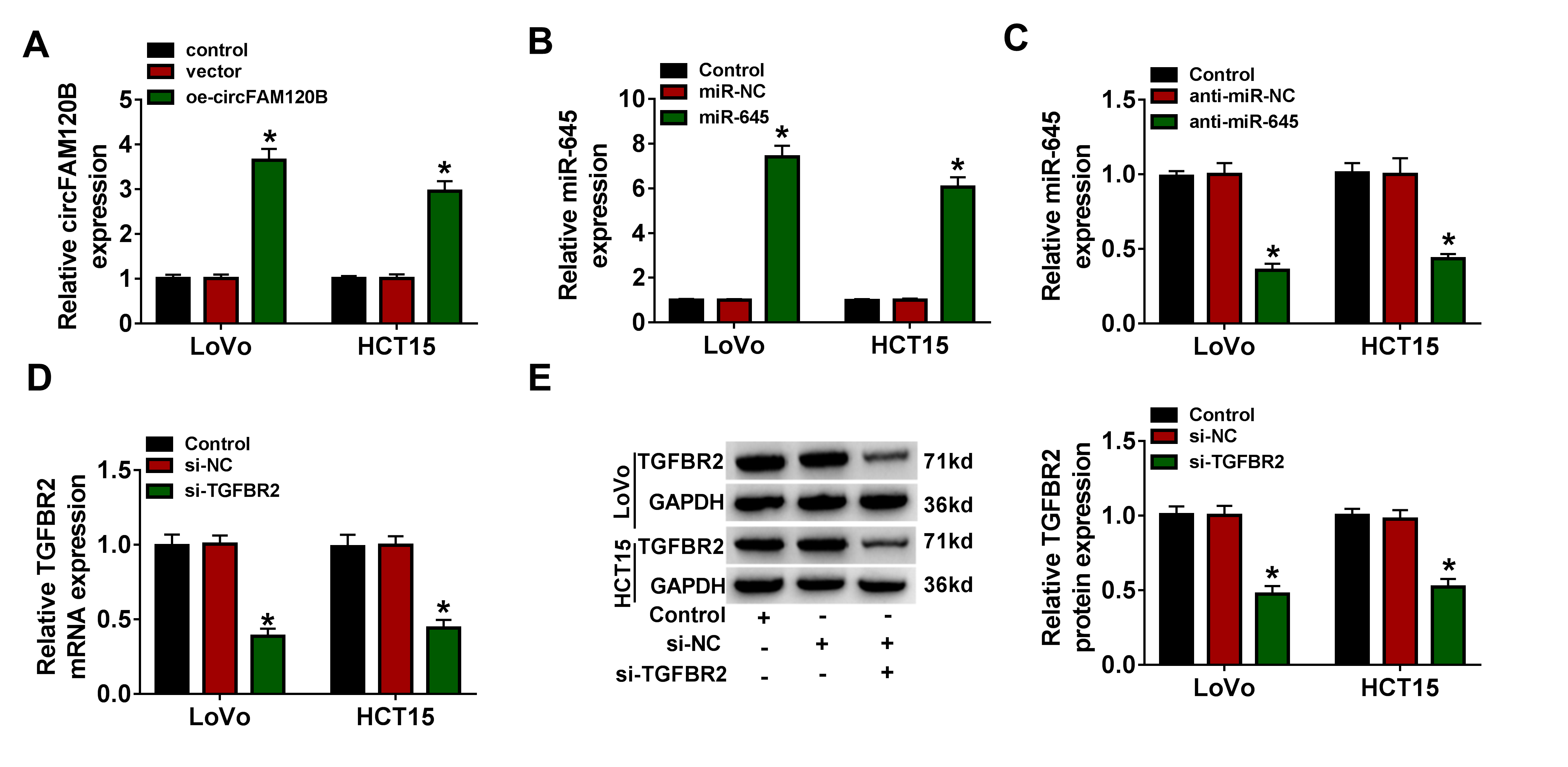

Supplement: Supplementary Figure 2 — The efficiency of different constructs. (A) The efficiency of oe-circFAM120B was checked by qRT-PCR. (B) The efficiency of miR-645 mimic was checked using qRT-PCR. (C) The efficiency of miR-645 inhibitor was checked using qRT-PCR. (D,E) The efficiency of si-TGFBR2 was checked using qRT-PCR and western blot. ∗P < 0.05. [file Image_2.TIF]

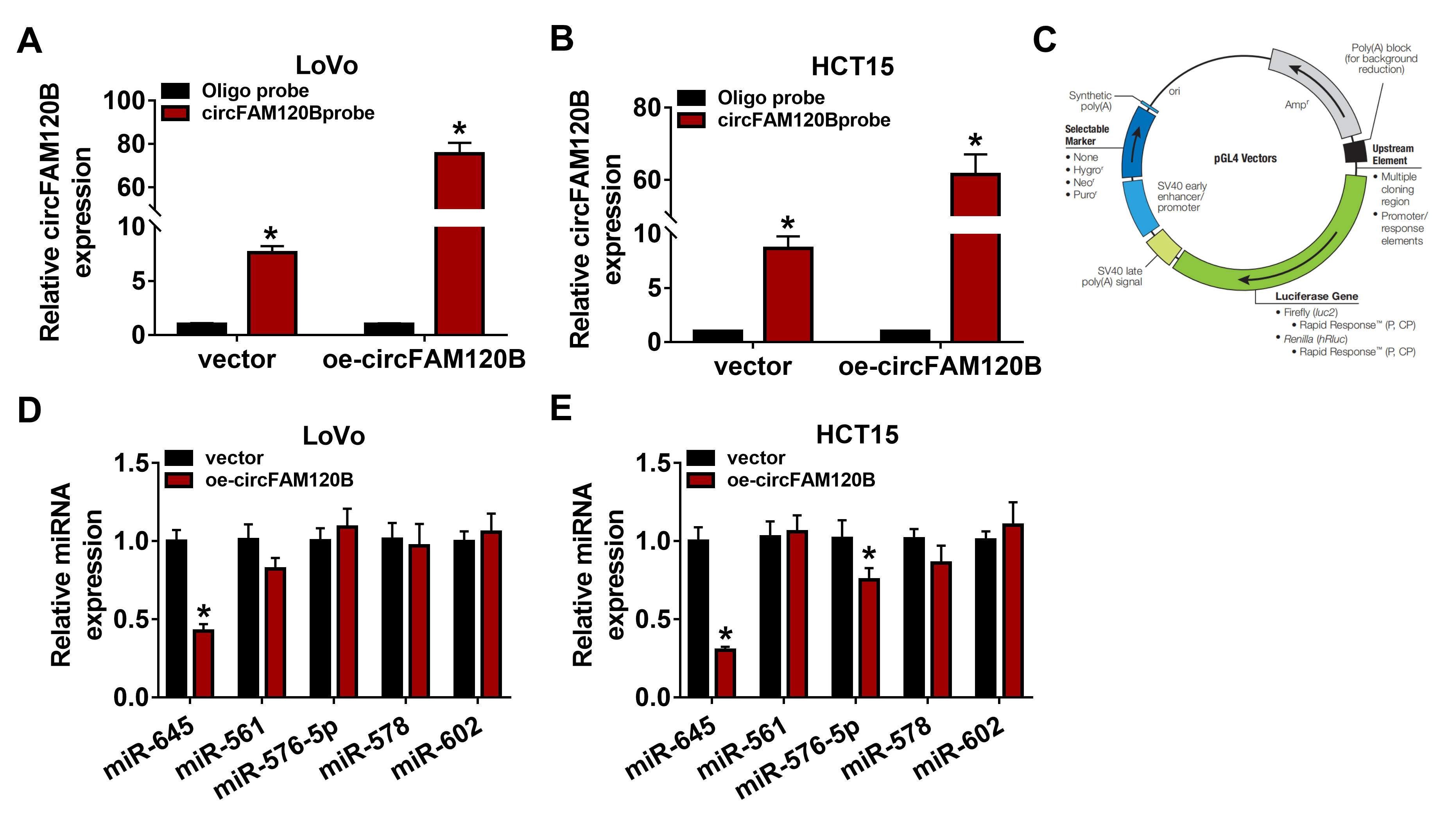

Supplement: Supplementary Figure 3 — The screening of miR-645. (A,B) The pull-down efficiency of circFAM120B probe was increased in LoVo and HCT15 cells with circFAM120B overexpression. (C) The information of luciferase reporter vector (PGL4). (D,E) The expression of putative target miRNAs of circFAM120B predicted by the bioinformatics tool was measured in LoVo and HCT15 cells with circFAM120B overexpression using qRT-PCR. ∗P < 0.05. [file Image_3.TIF]
